# Cuffless Blood Pressure Estimation Using Calibrated Cardiovascular Dynamics in the Photoplethysmogram

**DOI:** 10.3390/bioengineering9090446

**Published:** 2022-09-06

**Authors:** Hamed Samimi, Hilmi R. Dajani

**Affiliations:** School of Electrical Engineering and Computer Science, University of Ottawa, Ottawa, ON K1N 6N5, Canada

**Keywords:** cuffless blood pressure estimation, noninvasive blood pressure measurement, cardiovascular dynamics, photoplethysmogram (PPG), reflective pulse transit time (R-PTT), artificial neural network, blood pressure mathematical model

## Abstract

An important means for preventing and managing cardiovascular disease is the non-invasive estimation of blood pressure. There is particular interest in developing approaches that provide accurate cuffless and continuous estimation of this important vital sign. This paper proposes a method that uses dynamic changes of the pulse waveform over short time intervals and calibrates the system based on a mathematical model that relates reflective PTT (R-PTT) to blood pressure. An advantage of the method is that it only requires collecting the photoplethysmogram (PPG) using one optical sensor, in addition to initial non-invasive measurements of blood pressure that are used for calibration. This method was applied to data from 30 patients, resulting in a mean error (ME) of 0.59 mmHg, a standard deviation of error (SDE) of 7.07 mmHg, and a mean absolute error (MAE) of 4.92 mmHg for diastolic blood pressure (DBP) and an ME of 2.52 mmHg, an SDE of 12.15 mmHg, and an MAE of 8.89 mmHg for systolic blood pressure (SBP). These results demonstrate the possibility of using the PPG signal for the cuffless continuous estimation of blood pressure based on the analysis of calibrated changes in cardiovascular dynamics, possibly in conjunction with other methods that are currently being researched.

## 1. Introduction

Cardiovascular disease (CVD) is one of the major contributors to human mortality worldwide [1]. A primary risk factor for CVD is high blood pressure (BP) or hypertension, which is also called the silent killer because, in preliminary stages, it mostly develops with patients unaware of their condition [2]. Hypertension is not limited only to the older age group; 1 in 8 adults aged between 20 and 40 years suffers from high blood pressure [3]. The number of people affected by hypertension is expected to increase based on trends in lifestyle and behavior (such as low physical activity, poor eating habits, the high consumption of animal fat) and the lowering of the threshold for the diagnosis of hypertension [3]. In 2017, the American College of Cardiology and the American Heart Association introduced new definitions of hypertension, considering it present if the systolic blood pressure (SBP) or diastolic blood pressure (DBP) exceeds 130 mmHg or 80 mmHg, respectively. At the same time, an SBP between 120 and 129 mmHg is considered elevated [4].

People who are diagnosed with hypertension take medication in an effort to keep their BP under control. Any undiagnosed or untreated hypertension in early stages can potentially result in myocardial infarction, ischemic and hemorrhagic stroke, heart failure, chronic kidney disease, cognitive decline, and early death [5]. Blood pressure is a dynamic vital sign that varies over time due to age, physical activity, mental stress, etc. Therefore, the continuous monitoring of blood pressure could reveal information on these dynamic characteristics [6] and thus plays an important role in the diagnosis and effective management of hypertension [2]. Despite the importance of continuous blood pressure monitoring, at the present time, there are no consumer devices on the market that are medically approved for measuring blood pressure non-invasively and continuously [7]. Currently, the gold standard method for continuous BP monitoring is the direct (intra-arterial) method. This technique is mostly used in a clinical setting for patients with an unstable condition or patients who are under vasoactive treatments. While it allows for continuous BP measurement with a high accuracy, this method requires the invasive insertion of an arterial line into the patient’s artery [8]. Due to the setting limitation and the risk of severe bleeding upon accidental disconnection as well as infection for patients, this technique is not suitable for long-term BP monitoring [8]. Therefore, blood pressure is typically measured using non-invasive cuff-based methods which rely on the inflation and deflation of a cuff either manually or automatically. However, these methods cannot provide continuous monitoring, and applying pressure through a cuff wrapped around a limb can cause discomfort and potentially disturb sleep during nocturnal measurements [4]. Furthermore, the cuff-based method cannot be used on people with several pre-existing conditions such as lymphedema [9]. As a result, the development of cuffless BP measurement methods is in demand.

The most common physiological signals used in cuffless BP measurements are the electrocardiogram (ECG) and the photoplethysmogram (PPG). The ECG reflects the electrical excitation of the heart, while the PPG indicates blood volume changes in peripheral circulation [10]. These two signals are used in several of the major methods that are currently being researched for cuffless continuous blood pressure estimation, which are based on pulse transit time (PTT), pulse arrival time (PAT), pulse wave velocity (PWV), and pulse wave analysis [11].

PTT is defined as the time that the pressure wave takes to travel between two arterial sites [10], and it can be measured using photoplethysmogram (PPG) sensors placed at two sites on the body [6]. It can be calculated in different ways, such as the time delay between the proximal and distal PPG waveforms [6], the time difference from the mid-point of the falling edge of the proximal PPG to the peak of the peripheral PPG [12], or the time difference from the dicrotic notch of the proximal PPG to the peak of the peripheral PPG [13].

PAT is defined as the time interval between electrical activation of the heart and the arrival of the blood to the periphery from the aorta [14], and it is usually measured as the time interval between the R-peak in the ECG and the pulse waveform in the PPG recorded at a peripheral site [11]. Both PTT and PAT vary inversely with BP due to the physical properties of arteries [11]. It is also worth mentioning that many studies incorrectly refer to PAT as PTT, and the use of the true PTT is not greatly researched [6].

PWV is the speed of the pulse wave moving along the arterial vessels, and it is inversely related to PTT and PAT but is directly related to BP [15]. PWV is often used as the basis for cuffless methods to estimate blood pressure and is founded on the theory of fluid wave propagation through elastic pipes [16]. PWV can be measured using a few different methods such as high-fidelity pulse pressure measuring devices, Doppler, and phase contrast magnetic resonance imaging (MRI) [17]. However, the main concept behind this measurement is to calculate the velocity as the distance the pulse wave travels divided by the time of travel [11].

In addition to methods based on PAT, PTT, and PWV, there has been some work on BP estimation based on pulse wave analysis. In these methods, features are extracted from the pulse waveform, typically obtained with a single PPG sensor, which are then used in various machine learning estimation models. Most such features are generated from pulse morphology within individual beats [11]. In particular, data from a single PPG sensor have been used in some studies within a deep learning framework [18,19]. A detailed review of different PPG-based methods can be found in [20].

Some of the methods for the cuffless estimation of BP rely on mathematical models, which are usually established through the use of a transfer function [21], Windkessel models [22], the application of the Moens–Korteweg (M-K) equation, heuristic modeling with regression techniques, or predictive modeling with data-driven methods [23].

In previous work, we used a calibration-free method to estimate blood pressure using information from dynamic changes of the pulse waveform over brief time intervals [24]. In this work, we added a model-based calibration stage. To implement this process, we used data from a single PPG sensor to calculate reflective PTT (R-PTT) and calibrate a mathematical model derived from the Moens–Korteweg and Bramwell–Hill equations to estimate blood pressure values. The estimated values, along with some characteristics of cardiovascular dynamics expressed in the arterial pulse waveform oscillation, were then used to improve the cuffless blood pressure estimates. The latter was done because PTT by itself or features derived from the morphology of individual pulses have not yet been shown to be sufficient for accurate blood pressure estimation, and there is likely important information related to BP contained in the dynamics of the human cardiovascular system [25]. In this work, we use mean point-to-point pairing calibration (mPTP) to develop our model. This is a novel approach in model-based BP estimation based on reflective PTT. Furthermore, to the best of our knowledge, there has been no work done previously on combining R-PTT-based modeling and information from cardiovascular dynamics for the cuffless estimation of blood pressure.

## 2. Methods

**Figure 1:**
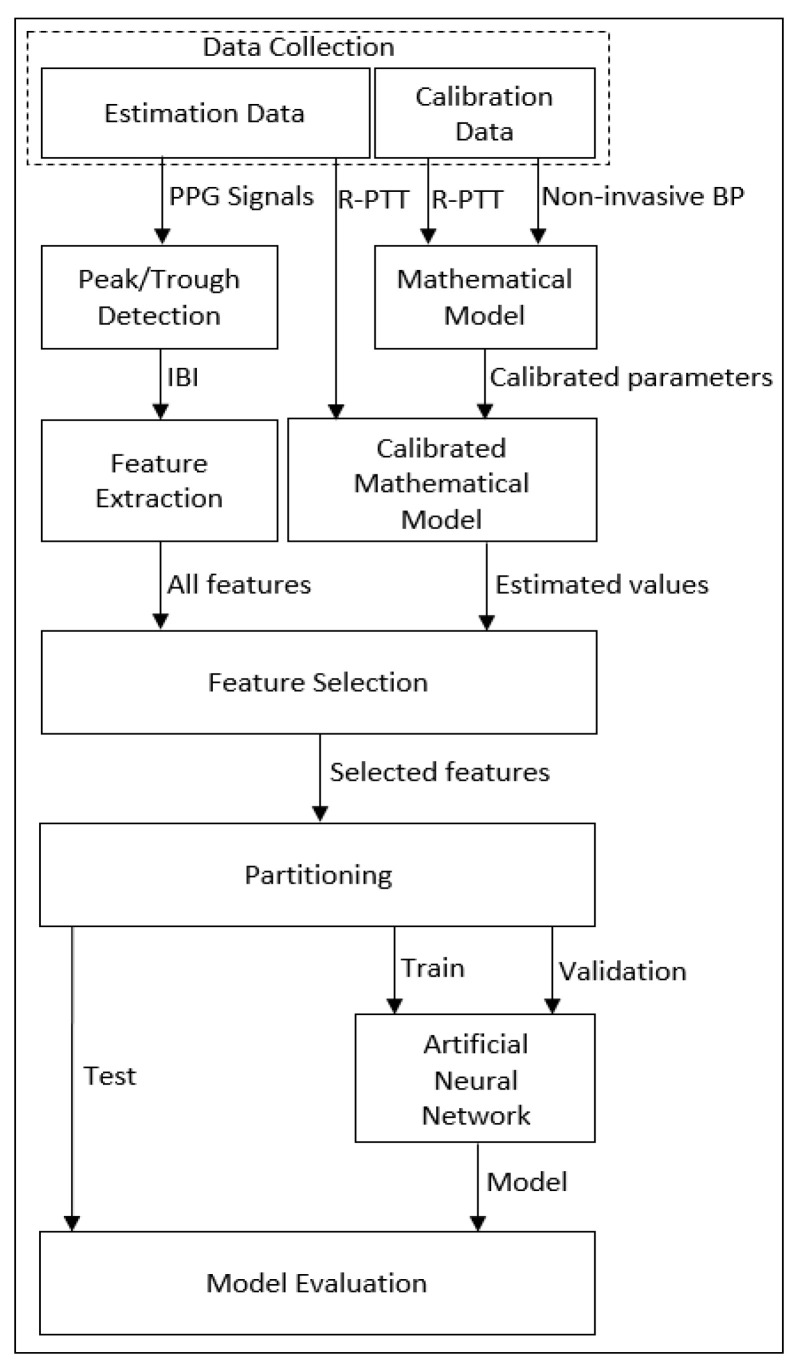
Block diagram of the method proposed in this paper for cuffless blood pressure estimation.

Figure 1 shows a high-level block diagram of the proposed methodology for cuffless blood pressure estimation, which uses information related to cardiovascular dynamics extracted from PPG signals along with a mathematical model based on Bramwell–Hill [26] and Moens–Korteweg equations [27].

### 2.1. Data Collection

In this study, the bio-signal data from the University of Queensland Vital Signs Dataset [28] is used. This dataset is the result of recording in four operating rooms from 32 patients, with a duration ranging from 13 min to 5 h and a median of 105 min. The 32 recorded cases consist of 25 under general anesthetics, 3 under spinal anesthetics, and 4 sedation cases. The unique characteristic of this dataset, which does not exist in data collected from intensive care units, is that it includes entire cases of patients undergoing anesthesia for surgery, which results in rapid and dynamic vital sign changes during the induction and emergence phases of anesthesia and surgery [28]. For all patients, ECG, PPG, and noninvasive arterial BP waveforms are recorded, while signals such as capnographs, airway flows, and others are collected on a case-by-case basis under the discretion of the anesthesiologist. The waveforms are sampled at 100 Hz.

In our work, we used only the PPG and noninvasive arterial BP signals from the dataset. Reference [28] does not specify the method used to collect the noninvasive arterial BP, but this signal is calibrated in mmHg. Of note, our method can make use of any calibrated BP measurements during an initial interval as inputs to the mathematical model (described below), including those obtained using a cuff-based method. We visually inspected all data and chose our dataset by selecting the ones with minimal or no interruption. As a result, our final selection consisted of data from 30 patients.

### 2.2. Peak/Trough Detection

To detect peaks and troughs of the PPG signals, we used a modified version of Pan and Tompkins’ QRS detection algorithm [29]. In this process, first, the PPG is filtered in two stages: a low-pass filter and a high-pass filter. The combination of these two filters forms a band-pass filter which reduces the effect of muscle noise, 60 Hz interference, and baseline wander. The filters used in this work are fourth-order low-pass with a cut-off frequency of 10 Hz and third-order Butterworth high-pass with a cut-off frequency of 0.05 Hz.

**Figure 2:**
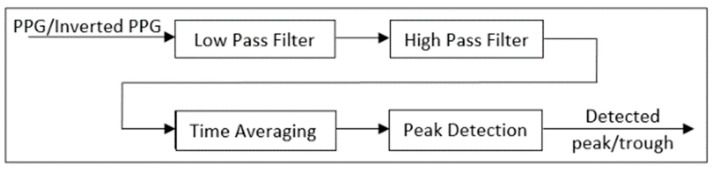
High-level block diagram of the peak/trough detection algorithm.

**Figure 3:**
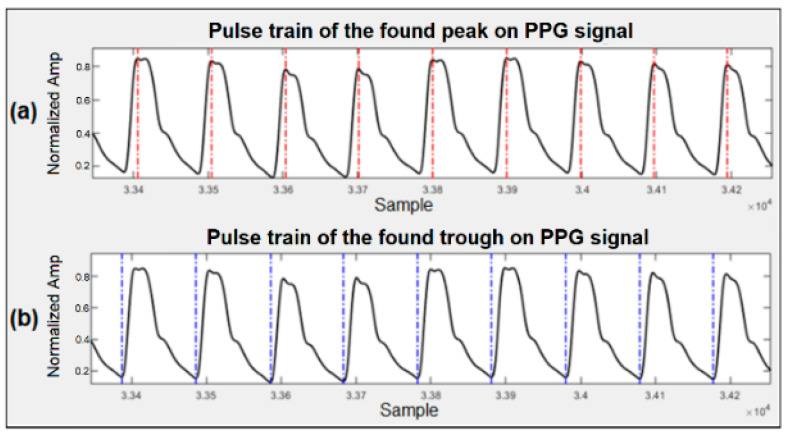
Detection of peaks and troughs in the PPG signal. (**a**) Detected peaks marked with dotted red lines. (**b**) Detected troughs marked with dotted blue lines.

After filtering, the signal goes through a moving average filter of length 38. A threshold is then set for the output of the moving average filter to determine the peaks of the signal based on the local maxima within a preset time interval. These maxima mark the locations of the arterial pulse waveform peaks or the PPG peaks. The troughs of the arterial pulse waveform or the PPG are detected by inverting the original signal and finding the peaks for the inverted waveform. Figure 2 shows the different stages of this process, and Figure 3 illustrates a sample of peak and trough detection for a typical PPG signal using the proposed algorithm.

### 2.3. Feature Extraction

We extracted features linked to cardiovascular dynamics from inter-beat intervals (IBIs) of the PPG signals through time domain, frequency domain, and nonlinear analysis. The analysis was performed on the last ten minutes of each of the thirty signals. For collecting IBI features, we used the open-source heart rate variability analysis software (HRVAS) [30]. The time series containing IBI are the inputs to the analyzer, and the extracted features are the outputs.

For the IBI time domain analysis, both statistical and geometric measurements are considered. Statistical measurements are calculated directly from the IBI series and include: the mean IBI, the standard deviation for the IBI series (SDANN), the pulse rate variability triangular index (PRVti), the triangular interpolation of IBI (TINN), the root mean square of successive differences of the IBI series, the number of successive differences that are greater than a user-defined threshold (NNx) in milliseconds (for our analysis, the threshold was set to 10 ms), and the percentage of NNx over the duration of the signal.

To compute SDANN, first, the IBI series is divided into an equal number of segments with no overlaps; then, the mean IBI for each segment is calculated, and the standard deviation of all the means is determined. The following equation represents the above steps for calculating SDANN [31].

(1) $$\mathrm{SDANN}=\sqrt{\frac{1}{M-1}\overset{M}{\underset{i=1}{\sum }}{\left[\mathrm{mean}\;\mathrm{IBI}\left(i\right)-\bar{\mathrm{mean}\;\mathrm{IBI}}\right]}^{2}}$$

In this equation, mean IBI(i)  represents the mean IBI value of the ith IBI segment, and M is the total number of segments.

**Figure 4:**
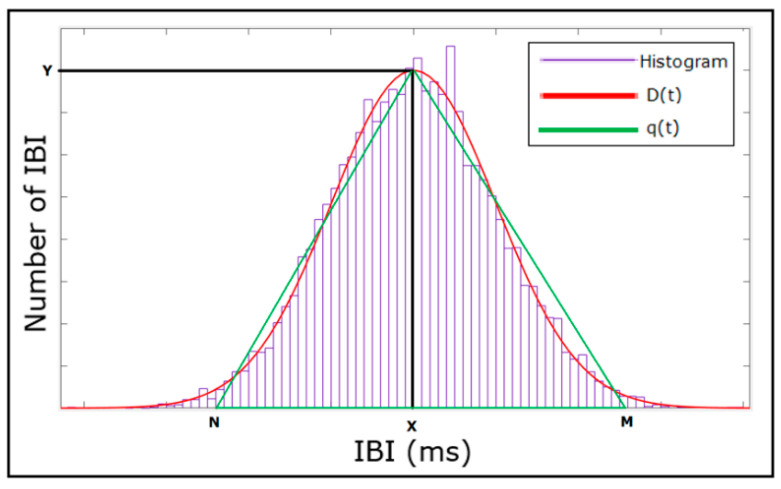
Histogram of a typical IBI time series. D(t) is a density distribution of IBI and q(t) is the triangular function fitted to D(t). Y is the maximum value of D(t), where t = x, and M and N are the other two vertices of the triangular function.

Geometrical measurements are calculated based on the IBI histogram [30]. The measured parameters are the pulse rate variability triangular index and the triangular interpolation of the IBI histogram. Figure 4 shows the histogram for a typical IBI time series with a density distribution of D(t). Y on the graph represents the maximum value for D(t) at t = x. The pulse rate variability triangular index is calculated by dividing the area integral of D(t) by the value of Y. If distribution D(t) is based on a discrete horizontal scale, the area integral is then the total number of IBIs, and the pulse rate variability triangular index (PRVti) can be calculated as:

(2) $$\mathrm{PRVti}=\frac{N_{\mathrm{IBI}}}{Y}$$

where $N_{\mathrm{IBI}}$ is the total number of IBIs and Y is the maximum value for D(t).

To obtain triangular interpolation of the IBI histogram, a triangular function q(t) is established in a way that the vertices of the triangle are Y, M, and N, where M and N are selected such that q(t) = 0 for M≤t≤N and by minimizing:

(3) $$\int_{0}^{+\infty }{\left(D\left(t\right)-q\left(t\right)\right)}^{2}\mathrm{dt}$$

The triangular interpolation of the IBI histogram (TINN) is then calculated as:

(4) Triangular interpolation of IBI=M− N

The IBI frequency domain analysis is performed by using the Lomb–Scargle periodogram (LSP) [32]. In the LSP method, the frequency spectrum is estimated by performing a least squares fitting of data by sinusoids. The LSP of non-uniformly sampled data is defined as:

(5) $$P_{\mathrm{LS}}\left(f\right)=\frac{1}{2\sigma^{2}}\left(\frac{{\left[\sum_{n=1}^{N}\left(X\left(t_{n}\right)-\;\bar{X}\;\right)\;\cos\left(2\pi f\left(t_{n}-\tau \right)\right)\right]}^{2}}{\sum_{n=1}^{N}\cos^{2}\left(2\pi f\left(t_{n}-\tau \right)\right)}+\frac{{\left[\sum_{n=1}^{N}\left(X\left(t_{n}\right)-\;\bar{X}\;\right)\;\sin\left(2\pi f\left(t_{n}-\tau \right)\right)\right]}^{2}}{\sum_{n=1}^{N}\sin^{2}\left(2\pi f\left(t_{n}-\tau \right)\right)}\right)$$

where X is a real valued data sequence of length N for arbitrary times $t_{n}$, $\bar{X}$ is the mean and σ is the variance of the time series, and τ is the frequency-dependent time delay defined as:

(6) $$\tau =\frac{\tan^{-1}\left(\left(\sum_{n=1}^{N}\sin\left(4{\pi \mathrm{ft}}_{n}\right)\right)/\left(\sum_{n=1}^{N}\cos\left(4{\pi \mathrm{ft}}_{n}\right)\right)\right)}{4\pi f}$$

to make the periodogram insensitive to the time shift.

In this method, the power spectral density of an IBI signal is calculated and divided into three sections labeled as very low frequency (VLF, 0.098–0.05 Hz), low frequency (LF, 0.05–0.15 Hz), and high frequency (HF, 0.15–0.5 Hz). The resistance to errors from data removal and resampling makes LSP a preferred method for power spectrum calculation for IBI signals [33].

A Poincaré plot, sample entropy, and detrended fluctuation analysis are used to analyze the nonlinear behavior of IBI signals. The Poincaré plot or return map is used to quantify self-similarity by mapping two consecutive IBIs in relation to each other [34]. An ellipse is fitted to this map, with the major and minor semi-axes of the ellipse referred to as SD2 and SD1, respectively. SD1 represents the standard deviation of instantaneous beat-to-beat variability, and SD2 characterizes the standard deviation of continuous beat-to-beat variability. These two axes are defined as

(7) $$\mathrm{SD}1=\frac{\sqrt{2}}{2}\;\mathrm{SD}\left(x_{n}-x_{n-1}\right)$$

(8) $$\mathrm{SD}2=\sqrt{2\mathrm{SD}{\left(x_{n}\right)}^{2}-\frac{1}{2}\mathrm{SD}{\left(x_{n}-x_{n-1}\right)}^{2}}$$

where $x_{n}$ and $x_{n-1}$ are two consecutive data points, and SD represents the standard deviation of the time series.

Sample entropy is a measure used to quantify signal complexity [35]. It is represented by SampEn(m.r,N) and is defined as the negative logarithm of the conditional probability that, for a dataset with N number of data points, if two sets of simultaneous data points of length equal to embedding dimension m  have a distance smaller than tolerance r, then two sets of simultaneous data points of length m+1 also have a distance smaller than r. A value of zero for the sample entropy indicates that the two consecutive sequences are identical, while a larger value represents higher complexity.

Detrended fluctuation analysis (DFA) [36] is another method that is used for the nonlinear analysis of IBI. DFA is a method to determine the statistical self-similarity of a signal and is based on the idea of the signal being similar to part of itself. For DFA processing, a bounded time series of IBIs is converted to an unbounded process through:

(9) $$y\left(k\right)=\overset{k}{\underset{i=1}{\sum }}\left[\mathrm{IBI}\left(i\right)-\bar{\mathrm{IBI}}\right]$$

where y(k) is cumulative sum or profile, IBI(i) is the ith inter-beat interval, and $\bar{\mathrm{IBI}}$ is the average inter-beat interval over the entire time series. The cumulative sum then is divided into segments of length N, and the square error for each part is minimized to fit a local least square straight-line to the data and define the local trend $y_{n}\left(k\right)$. The fluctuation is then considered as the root mean square deviation from the trend:

(10) $$F\left(n\right)=\sqrt{\frac{1}{N}\overset{N}{\underset{k=1}{\sum }}{\left(y\left(k\right)-y_{n}\left(k\right)\right)}^{2}}$$

where N represents the window size.

The above-mentioned process is repeated over different ranges of window sizes; then, the linear relationship between F(n) and n is plotted to obtain the scaling exponents $\alpha_{1}$ (short term scaling) and $\alpha_{2}$ (long term scaling) for the inter-beat interval time series. Table 1 summarizes extracted IBI features for our analysis.

### 2.4. Mathematical Model

To create a mathematical model for the estimation of blood pressure, we used the Bramwell–Hill and Moens–Korteweg equations. The blood pressure can be calculated based on the theory of pulse wave velocity using the following equation:

(11) $$\mathrm{PWV}=\sqrt{\frac{V}{\rho }\frac{∆P}{∆V}}$$

where V is the volume of blood in the artery, ρ is the blood density, ∆P is the difference between SBP and DBP, and ∆V is the corresponding blood volume change [26]. Since, for each individual, the blood density, the blood volume in the artery, and the change in the blood volume are near constant, (11) can be simplified as:

(12) $$\mathrm{SBP}-\mathrm{DBP}=\frac{\rho ∆V}{V}{\left(\mathrm{PWV}\right)}^{2}=\frac{\rho ∆V}{V}{\left(\frac{L}{\mathrm{PTT}}\right)}^{2}=K_{a}\frac{1}{{\mathrm{PTT}}^{2}}$$

where PTT is the pulse transit time it takes the pressure wave to travel between two arterial sites separated by a distance L, and $K_{a}$ is a parameter that needs to be calibrated for an individual by experiment [13].

In this study, we used a particular type of PTT called reflective PTT (R-PTT), whose main advantage is that it can be determined from the PPG signal obtained with a single optical sensor placed on the skin at a peripheral site, such as at the wrist. R-PTT is the duration that the pulse wave takes to travel from the radial artery to the end of the limb and reflect back to the radial artery again [13], and it can be measured by calculating the duration between the first and the second peaks of a single PPG pulse [37]. Substituting R-PTT in (12), we can obtain the SBP value using:

(13) $$\mathrm{SBP}=\mathrm{DBP}+K_{a}\frac{1}{R-{\mathrm{PTT}}^{2}}$$

PWV can also be measured using the Moens–Korteweg equation [27]:

(14) $$\mathrm{PWV}=\sqrt{\frac{E_{\mathrm{in}}h}{2\rho r}}$$

where $E_{\mathrm{in}}$ is the incremental elastic modulus of the artery, h is the thickness of the artery, r is the radius of the artery, and ρ is the density of the blood. Based on the experimental results obtained by [38], the elastic modulus of an artery can be represented as [13]:

(15) $$E_{\mathrm{in}}=E_{0}e^{\gamma \times \mathrm{MBP}}$$

where $E_{0}$ is the elastic modulus at zero pressure and γ is the coefficient depending on the particular vessel. The values of these two parameters are taken as 1428.7 and 0.031, respectively, which are the average values obtained in the study carried out by [38] for the brachial artery and have been used in a number of studies [13,39,40]. MBP is the mean blood pressure and can be derived by:

(16) $$\mathrm{MBP}\equiv \frac{1}{3}\mathrm{SBP}+\frac{2}{3}\mathrm{DBP}=K_{b}+\frac{2}{0.031}\ln\left(\frac{K_{c}}{R-\mathrm{PTT}}\right)$$

while $K_{b}$ and $K_{c}$ are parameters that need to be calibrated for an individual by experiment [13].

By substituting (13) into (16), the DBP value can be calculated using:

(17) $$\mathrm{DBP}=K_{b}+\frac{2}{0.031}\ln\left(\frac{K_{c}}{R-\mathrm{PTT}}\right)-\frac{K_{a}}{3{\left(R-\mathrm{PTT}\right)}^{2}}$$

**Figure 5:**
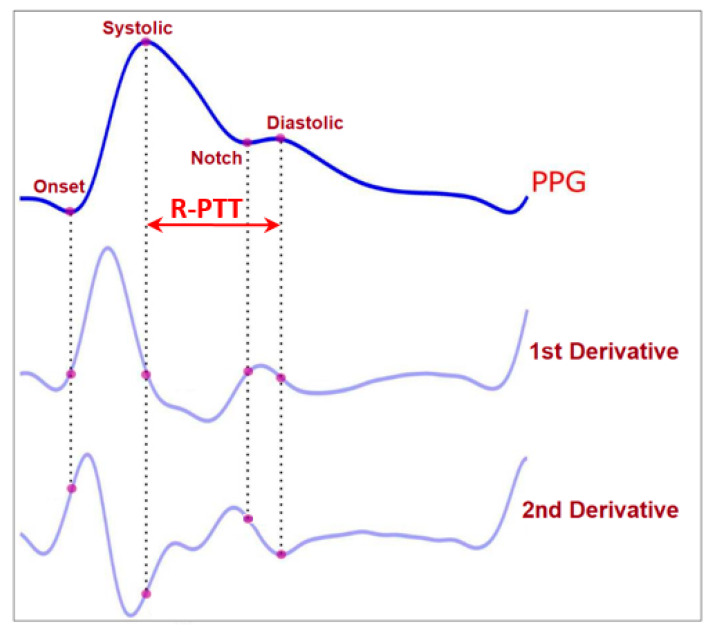
Illustration of the PPG signal and its first and second derivatives. The second derivative can locate the peak of the reflected wave (diastolic peak). The difference between the systolic peak and diastolic peak, in time, represents the reflective PTT (R-PTT) indicated by the horizontal line in the figure. (Adapted with modification from [41].)

The peak of the reflected wave can be located using the second derivative of the PPG signal (diastolic peak) [41]. The separation between the systolic and diastolic peaks on the time axis marks the reflective PTT [40]. Figure 5 shows the PPG waveform, its first and second derivatives, and the R-PTT.

An open-source software, PulseAnalyse [42], was used in this work to compute the R-PTT values. In previous works, where the reflective pulse transit time was used to estimate blood pressure [13,39,40], the R-PTT was utilized in the context of one point-to-point (oPTP) calibration. However, studies that were conducted on PTT-based BP estimation have shown that the oPTP method is not robust [43]. One way to make it more robust is to use mean point-to-point pairing calibration (mPTP) [43], which is what we did in this work. We calculated the average R-PTT over the first 30 s of the waveform pulse (i.e., mPTP) instead of in a single pulse, in addition to the average values of non-invasive SBP and DBP during this interval, and used this information to calibrate the $K_{a},\;K_{b},$ and $K_{c}$ parameters in our model for each individual using (13) and (17).

We then considered the last ten minutes of the dataset for each of the 30 patients to estimate the blood pressure using the developed mathematical model. Using this segment of the signals for estimation gave us the most separation from the calibration interval and thus provided a more realistic performance result of the model for real applications. For the estimation segment (last ten minutes of the waveform), we substituted the earlier calculated and calibrated parameters ($K_{a},{\;K}_{b},$ and $K_{c}$ from the first 30 s of the waveforms) and R-PTT values using the mPTP method (from the last 10 min of the waveforms) into (13) and (17) and calculated the SBP and DBP values for this segment. These model-based values of SBP and DBP obtained based on the initial calibration are in turn fed as features into the machine learning model, as described below.

### 2.5. Feature Selection

We used wrapper subset evaluation with the forward greedy stepwise search method [44] to determine two separate feature sets. For the first set, we only used features from cardiovascular dynamics that were generated using the IBI series. This provided us with four features for the estimation of systolic blood pressure (NNx, α1, LF, and HF) and five features for the estimation of diastolic blood pressure (SDNN, RMSSD, SD1, LF, and α1). These are the feature sets that were used with the Artificial Neural Network model to generate an estimation baseline. The estimation result from this baseline (cardiovascular dynamics features) is compared with the result obtained from the second set of the features to investigate the effect of incorporating the mathematical model on the estimation accuracy. For the second set, we used the estimated blood pressure values from the mathematical model in addition to the features from cardiovascular dynamics. This provided us with three features for the estimation of systolic blood pressure (SampleEn, α1, and systolic estimation from the mathematical model) and also three features for the estimation of diastolic blood pressure (SampleEn, HF, and diastolic estimation from the mathematical model).

### 2.6. Partitioning

In order to partition data to obtain the baseline estimation, we used the leave-one-out method. The same method was also considered to evaluate the performance of the new estimator that incorporates the mathematical model. The collected data were from 30 patients. For both feature sets, all of the above-mentioned features from one patient were set aside to be used as the test data, while the remaining features were split into 85% training and 15% validation. This process was repeated 30 times to cover the entire dataset. To avoid overfitting, validation data with the early stopping technique were used [45].

### 2.7. Artificial Neural Network

An Artificial Neural Network (ANN) from the Deep Learning Toolbox version 13.0 in Matlab R2019b was used for regression. An ANN with a two-layer feed-forward network structure, a sigmoid layer followed by a linear output layer, was used. We tested different numbers of neurons to find the best structure for the network and chose a hidden layer with ten neurons based on the performance. To train the network, we used a Bayesian Regularization backpropagation algorithm and fixed the structure of the network prior to applying it to the test data. A separate network was trained for each of the two feature sets for SBP and DBP.

### 2.8. Model Evaluation

The estimation performance was evaluated based on the mean error (ME), the mean absolute error (MAE), and the standard deviation of error (SDE) obtained with the test data. The ME and MAE are calculated using the following equations:

(18) $$\mathrm{ME}=\frac{\sum_{i=1}^{n}y_{i}-x_{i}}{n}$$

(19) $$\mathrm{MAE}=\frac{\sum_{i=1}^{n}\left|y_{i}-x_{i}\right|}{n}$$

where $y_{i}$ is the prediction and $x_{i}$ is the true value for the SBP or DBP from the dataset. The true values are determined by averaging the SBP or DBP values that were measured non-invasively over the whole 10 min test interval.

The SDE was calculated based on the following equation:

(20) $$\mathrm{SDE}=\sqrt{\frac{\sum_{i=1}^{n}{\left|e_{i}-\;\bar{e}\;\right|}^{2}}{n}}$$

where $e_{i}$ is the error between the prediction and the true value ($e_{i}=y_{i}-x_{i}$) for each estimation, and $\bar{e}$ is the average of $e_{i}$.

### 2.9. Sensitivity Analysis of the Mathematical Model

Sensitivity analysis (SA) predicts the level of sensitivity of a model output to changes in parameter values [46]. Models with a high uncertainty and high sensitivity may experience a large variation in the output with a small change in the inputs [46]. SA is used to evaluate the relative importance of each input parameter and rank the model parameters from most to least influential [46]. In general, SA methods can be divided into local and global methods. Local SA is usually derivative-based and works by changing one variable at a time while keeping all the other variables constant and measuring changes in the output. This method is relatively simple to use; however, it provides information only at the central point and not the whole parameter space [46]. On the other hand, global SA covers the whole input parameter space, since all the input parameters are changed together, but at the expense of a higher computational cost [47].

In this work, we used an open-source toolbox in Matlab called Sensitivity Analysis For Everyone (SAFE) [48] to analyze the sensitivity of the calibration parameters in our mathematical model. This toolbox takes advantage of variance-based sensitivity analysis in order to determine the global SA.

For a generic model, $Y=f\left(X_{1},{\;X}_{2},{\;X}_{3},\;\ldots ,{\;X}_{k}\right),$ where Y is a scalar, a variance-based first-order effect for a generic factor $X_{i}$ can be shown as:

(21) $${\mathrm{Var}}_{X_{i}}(E_{X_{\sim i}}\left(Y|X_{i}\right))$$

where ${\mathrm{Var}}_{X_{i}}$ is the variance taken over all possible values of $X_{i}$, $X_{i}$ is the i-th factor, $X_{\sim i}$ is the matrix of all factors except $X_{i}$, and $E_{X_{\sim i}}$ is the expected value of Y over all possible values of $X_{\sim i}$. The first-order sensitivity that estimates the single contribution of each input parameter on the output variance and sensitivity can be measured as:

(22) $$S_{i}=\frac{{\mathrm{Var}}_{X_{i}}(E_{X_{\sim i}}\left(Y|X_{i}\right))}{\mathrm{Var}\left(Y\right)}$$

where $S_{i}$ is a normalized index, as ${\mathrm{Var}}_{X_{i}}(E_{X_{\sim i}}\left(Y|X_{i}\right))$ varies between zero and Var(Y). The total sensitivity index that measures the total contribution of each input parameter to the output variance and sensitivity can be calculated as:

(23) $$S_{\mathrm{Ti}}=\frac{E_{X_{\sim i}}({\mathrm{Var}}_{X_{i}}\left(Y|X_{\sim i}\right))}{\mathrm{Var}\left(Y\right)}=1-\frac{{\mathrm{Var}}_{X_{\sim i}}(E_{X_{i}}\left(Y|X_{\sim i}\right))}{\mathrm{Var}\left(Y\right)}$$

where $S_{\mathrm{Ti}}$ is the total sensitivity index and ${\mathrm{Var}}_{X_{\sim i}}$ is the variance taken over all possible values of $X_{\sim i}$ [47].

### 2.10. Computational Complexity

Table 2 summarizes the computational complexity analysis that was performed on every step of this work using the Matlab profiling capability. The computational complexity was determined based on the execution time and the amount of allocated memory for each part.

It is to be noted that, although training the model can be somewhat time-consuming, testing is not, especially because, in a real-world application, testing would be done on a single set of measurements and not 30, as was done here (i.e., the execution time would be divided by 30).

## 3. Results

In this work, we proposed a model-based approach to calibrate estimates of blood pressure using PPG signals. In this process, we estimated both systolic and diastolic blood pressure with the 30 patients using the following three methods:

- Information from cardiovascular dynamics was used to estimate blood pressure. This is a calibration-free method that we developed in our previous work [24]. The blood pressure estimation results based on this method are shown in Table 3. This table shows the error for the SBP and DBP attained using an ANN model on five extracted IBI features for DBP and four extracted IBI features for SBP;
- Calibrated mathematical model to estimate blood pressure, as was described in the methodology section. The blood pressure estimation based on this method is presented in Table 4. This table shows the error for the SBP and DBP obtained using this methodology;
- Information from cardiovascular dynamics, in addition to the calibrated mathematical model, is used for blood pressure estimation. The estimation results using this method are shown in Table 5. This table shows the error for the SBP and DBP obtained through an ANN model and based on three extracted IBI features for both SBP and DBP. This result can also be evaluated in reference to the IEEE 1708-2014 Standard for Wearable Cuffless Blood Pressure Measuring Devices [49]. This standard requires a number of conditions to be met and includes grading of the devices based on the obtained MAE. Solely based on the MAE criteria of the standard, our proposed method achieves a passing grade of A for DBP.

**Figure 6:**
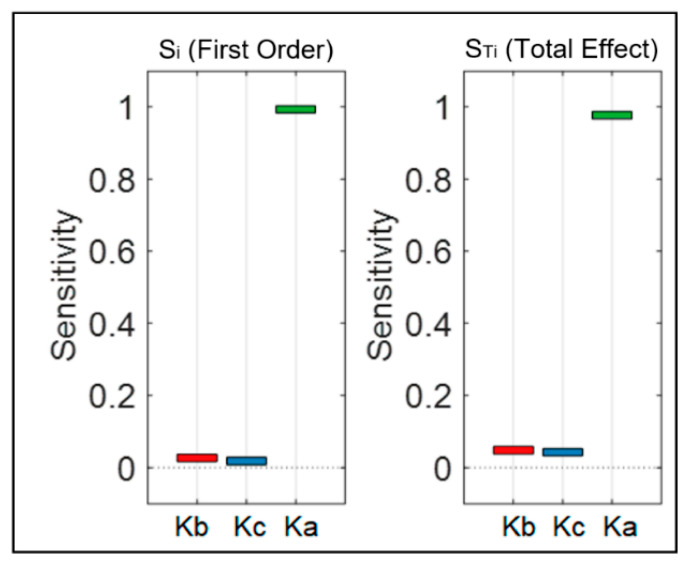
Sensitivity ranking for the SBP model. $S_{i}$ and $S_{\mathrm{Ti}}$ show the results for first-order sensitivity and total effect sensitivity, respectively. Red, blue and green bars correspond to $K_{b}$, $K_{c}$ and $K_{a}$ parameters respectively.

**Figure 7:**
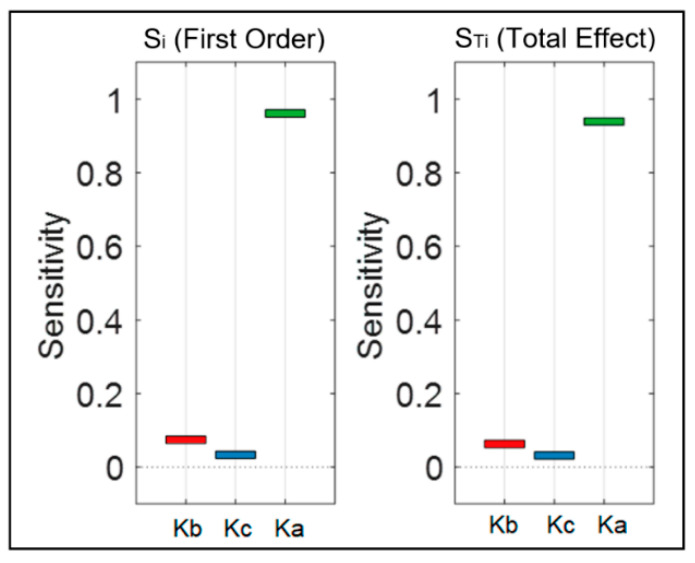
Sensitivity ranking for the DBP model. $S_{i}$ and $S_{\mathrm{Ti}}$ show the results for first order sensitivity and total effect sensitivity, respectively. Red, blue and green bars correspond to $K_{b}$, $K_{c}$ and $K_{a}$ parameters respectively.

We also performed sensitivity analysis for the proposed mathematical model to determine the stability of the model and evaluate the relative importance of each of the calibration parameters in the system. This analysis provided a ranking for the model parameters based on their influence on the system. Figure 6 shows the ranking result from the sensitivity analysis for the SBP model, and Figure 7 presents the ones for the DBP model. Rankings are based on first-order sensitivity indices, $S_{i}$ (contribution of each individual input to changes in the model output), and total effect sensitivity indices, $S_{\mathrm{Ti}}$ (the first-order sensitivity plus all the interactions involving that parameter). For both the systolic and diastolic models, $K_{a}$ and $K_{c}$ were the most and the least influential parameters, respectively. Therefore, according to the results from the sensitivity analysis, the $K_{a}$ parameter is mainly responsible for the variation in model predictions. Since the mathematical model was found to be sensitive to this parameter, effort should be made in carefully choosing its value during experimentation.

## 4. Discussion

The results of this research indicate that the proposed approach based on IBI dynamics over short intervals in conjunction with a mathematical calibration model using only a single photoplethysmogram signal could be used for cuffless blood pressure estimation. This approach may be used on its own or as a complement to other cuffless BP estimation methods. The advantage of this method is that, since the IBI dynamics of PPG signals rely only on the timing variation between the peaks or troughs of the signals, it is likely less sensitive to changes in sensor placement or to different skin colors. This may make blood pressure estimation using this method more favorable compared to methods, including those based on deep learning, that rely on within-beat PPG pulse morphology.

In this work, we looked into both a calibrated model as well as a calibration-free model for the cuffless estimation of BP. We collected 16 features from PPG IBI variability which were examined in our previous work [24]. We used wrapper subset evaluation with the forward greedy stepwise search technique to select a subset of these features as inputs to the ANN. For the calibration-free approach, five features for DBP estimation and four for SBP estimation were selected.

We also used a mathematical model for calibration based on the reflective PTT and initial non-invasive blood pressure measurements. Sensitivity analysis and parameter ranking showed that one of the parameters was dominant in terms of the sensitivity of the model to it. Considering this model, we selected three features to be used with the ANN for both SBP and DBP estimation using the wrapper subset evaluation with the forward greedy stepwise search method. It is seen that adding the mathematical calibration model led to a substantial reduction in most of the error measures, particularly the critical measures of SDE and MAE. These measures were reduced by 45% and 42% for SBP and 36% and by 35% for DBP, respectively.

The lower errors found for DBP vs. SBP may be partly explained by the strong relation that has been found between HRV indices and both SBP and DBP in females, while for males, there was no relation between these indices and SBP [50]. For the dataset that we used, there was no information provided regarding the gender composition; however, it is safe to assume that the number of male and female participants is likely to be close. Therefore, this could result in BP estimation based on IBI dynamics that is less accurate for SBP than for DBP. In addition, [51] found that changes in IBI are more clearly present in DBP compared to SBP. Therefore, the closer relationship between IBI dynamics and DBP could have contributed to the higher estimation accuracy of DBP.

In our previous work, we estimated blood pressure based on features from cardiovascular dynamics without calibration [24]. In the current study, features from cardiovascular dynamics were also considered for BP estimation; however, a mathematical calibration model was also added to investigate if it provided an improvement in performance. The selected features related to IBI dynamics in the two studies were different for both SBP and DBP estimation. In the previous study, five IBI-related features for the estimation of SBP (mean IBI, NNx, pNNx, SD2, and α1) and six features for the estimation of DBP (mean IBI, NNx, pNNx, PRVTi, SampleEn, and the IBI ratio of LF/HF) were selected, while in this work, we ended up using four features for the estimation of SBP (NNx, α1, LF, and HF) and five features for the estimation of DBP (SDNN, RMSSD, SD1, LF, and α1). The discrepancy in IBI-related feature selection may be due to the different target data in the two studies. In our previous work, the target for the estimation model was the invasive arterial blood pressure, whereas, in this study it was noninvasive arterial blood pressure. As noted in the literature, not only are there differences in BP readings between invasive and noninvasive methods, but substantial differences in BP measurements are also observed when different devices are used [52]. Another reason for the divergence in feature selection could be due to the choice of datasets. In our previous study, the dataset was collected in the intensive care unit (ICU), where the patient was possibly under the influence of medications or other interventions that could cause abnormal blood pressure dynamics. Additionally, some medications such as inotropes could result in BP measurement differences between invasive and noninvasive methods, and the difference increases with the amount of medication used [53]. We used signals from patients undergoing anesthesia for surgery, which results in rapid and dynamic vital sign changes during the induction and emergence phases of anesthesia. This study also had some limitations. First, some useful information such as the age, height, weight, and gender of the participants was missing from the dataset. These basic variables could provide valuable information for the estimation process. Second, the quality of the PPG signals was inspected manually, which is not an ideal practice in real-life scenarios. An automated replacement for this step that evaluates the quality of signals in a preprocessing stage would be useful. Third, the loss of blood volume during surgical procedures was not recorded. This would be of interest to us since our mathematical model assumes a near constant blood volume in arteries, and this could be affected by blood loss. Fourth, the size of the dataset imposed a limitation on providing a separate and independent test dataset to validate the accuracy of the model. A larger database would provide the flexibility to set aside a portion of the data solely for testing purposes and offer a larger training set for the network that can ultimately increase the accuracy of the estimation, as was found in some previous studies such as the ones in [18] and [19], where a larger dataset was used. However, there could be other reasons behind their reported better performance, such as:

- Reference blood pressure values were taken invasively, whereas, in our study, these values are collected through noninvasive methods.
- In [18], because of the large dataset, the authors could afford to apply a BP range constraint, meaning that, whenever the output was beyond a certain threshold, it was eliminated and not considered for performance evaluation.
- The input in those studies was based on segmented windows of the collected data with overlaps. This could cause the network to be exposed to the test data during training and result in a higher reported accuracy.

Regardless of these limitations, we showed that changes in IBI dynamics extracted from the photoplethysmogram (PPG) can be used to estimate BP. The estimation accuracy was further improved with the use of a mathematical calibration model. Even greater improvement might be achieved by combining the method proposed in this study with other approaches for cuffless BP estimation.

## 5. Conclusions and Future Work

The concept of blood pressure estimation based solely on cardiovascular dynamics is different from current prevalent approaches that use pulse transit time or pulse morphology within individual beats. It is understood that the cardiovascular dynamics carry useful information that can help to recognize conditions such as hypertension [54]. This was demonstrated in the study by [51], where it was concluded that there are differences in the short-term oscillation in blood pressure (BP) between normotensive, borderline hypertensive and hypertensive individuals [51]. Inspired by the above findings, our proposed method to estimate blood pressure is to use information from dynamic changes (that are collected over short intervals of a few minutes) and incorporate a mathematical calibration model based on the initial measured values of blood pressure.

In this paper, we compared the estimated values for both SBP and DBP using a calibration-free model and the proposed calibrated model. The overall estimation results were in line with the expectation that the cardiovascular dynamics contain valuable information for the estimation of blood pressure. In addition, the result also showed that the calibration stage improved the accuracy in both SBP and DBP estimation. However, to further improve cuffless BP accuracy measurement, in future work, this approach may be combined with widely used methods based on the pulse morphology within beats or the pulse transit time.

## Figures and Tables

**Table 1:** List of considered features related to the cardiovascular dynamics used in this study.

Type of Signal Used	Measure	Description
IBI Time-Domain	Mean IBI	Mean of IBI
IBI Time-Domain	SDANN	Standard deviation of IBI
IBI Time-Domain	RMSSD	Root mean square of successive differences of the IBI series
IBI Time-Domain	NNx	Number of successive IBIs that are longer than a user-defined threshold in milliseconds
IBI Time-Domain	pNNx	Percentage of NNx over the duration of the signal
IBI Time-Domain	PRVti	Pulse rate variability triangular index
IBI Time-Domain	TINN	Triangular interpolation of the IBI histogram
IBI Frequency-Domain	VLF	Power of very-low-frequency band
IBI Frequency-Domain	LF	Power of low-frequency band
IBI Frequency-Domain	HF	Power of high-frequency band
IBI Frequency-Domain	LF/HF	Ratio between low- and high-frequency band powers
IBI Nonlinear-Domain	SD1	Standard deviation of instantaneous beat to beat variability (Poincaré plot)
IBI Nonlinear-Domain	SD2	Standard deviation of continuous beat to beat variability (Poincaré plot)
IBI Nonlinear-Domain	SampEn	Sample entropy
IBI Nonlinear-Domain	$\alpha_{1\;}$	Short-term fluctuation slope in Detrended Fluctuation Analysis
IBI Nonlinear-Domain	$\alpha_{2\;}$	Long-term fluctuation slope in Detrended Fluctuation Analysis

**Table 2:** Computational complexity based on execution time and memory allocation. The CPU clock speed during this process was 2.501 GHz. The execution time was measured with a precision of 1.00 × 10^−7^ s. The data for the peak/trough detection, feature extraction, and mathematical model are from a single sample file. These values are the same for both the training and test data. For ANN, the numbers correspond to a set of 30 measurements (our complete dataset).

	Total Execution Time (s)	Total Allocated Memory (Mbytes)
Peak/trough detection	183.72	941.66
Feature extraction	59.80	261.33
Mathematical model	218.30	2051.61
ANN training	509.31	8.48
ANN testing	11.65	4.63

**Table 3:** Blood pressure estimation performance using IBI features from 30 PPG waveforms. Five features are used for DBP and four are used for SBP. Estimation is carried out using the ANN described in the text, and the results are averaged over the 30 patients.

Dynamics			
	ME (mmHg)	SDE (mmHg)	MAE (mmHg)
Diastolic BP	0.14	10.97	7.54
Systolic BP	−0.39	22.16	15.26

**Table 4:** Blood pressure estimation performance using reflective PTT in a calibrated mathematical model with 30 PPG waveforms. The results are averaged over the 30 patients.

Modeling			
	ME (mmHg)	SDE (mmHg)	MAE (mmHg)
Diastolic BP	0.45	8.36	5.47
Systolic BP	3.18	12.49	9.11

**Table 5:** Blood pressure estimation performance using IBI features and estimation from a calibrated mathematical model from 30 PPG waveforms. Three features are used for SBP and DBP. Estimation is carried out using the ANN described in the text, and the results are averaged over the 30 patients.

Dynamics + Modeling			
	ME (mmHg)	SDE (mmHg)	MAE (mmHg)
Diastolic BP	0.59	7.07	4.92
Systolic BP	2.52	12.15	8.89

## Data Availability

In this study, the bio-signal data from the University of Queensland Vital Signs Dataset are used [28].

## References

[1] Barquera S., Pedroza-Tobias A., Medina C., Hernandez-Barrera L., Bibbins-Domingo K., Lozano R., Moran A.E. (2015). Global overview of the epidemiology of atherosclerotic cardiovascular disease. Arch. Med. Res..

[2] Kachuee M., Kiani M.M., Mohammadzade H., Shabany M. (2017). Cuffless blood pressure estimation algorithms for continuous health-care monitoring. IEEE Trans. Biomed. Eng..

[3] Hinton T.C., Adams Z.H., Baker R.P., Hope K., Paton J., Hart E., Nightingale A. (2020). Investigation and treatment of high blood pressure in young people. Hypertension.

[4] Tamura T. (2021). Cuffless blood pressure monitors: Principles, standards and approval for medical use. IEICE Trans. Commun..

[5] Bayrak D., Tosun N. (2018). Determination of nursing activities for prevention of heart attack and stroke in hypertension patients. Int. J. Caring Sci..

[6] Le T., Ellington F., Lee T.Y., Vo K., Khine M., Krishnan S.K., Dutt N., Cao H. (2020). Continuous non-invasive blood pressure monitoring: A methodological review on measurement techniques. IEEE Access.

[7] Baker S., Xiang W., Atkinson I. (2021). A hybrid neural network for continuous and non-invasive estimation of blood pressure from raw electrocardiogram and photoplethysmogram waveforms. Comput. Methods Programs Biomed..

[8] Esmaelpoor J., Moradi M.H., Kadkhodamohammadi A. (2020). A multistage deep neural network model for blood pressure estimation using photoplethysmogram signals. Comput. Biol. Med..

[9] Cemal Y., Pusic A., Mehrara B.J. (2011). Preventative measures for lymphedema: Separating fact from fiction. J. Am. Coll. Surg..

[10] Esmaelpoor J., Moradi M.H., Kadkhodamohammadi A. (2021). Cuffless blood pressure estimation methods: Physiological model parameters versus machine-learned features. Physiol. Meas..

[11] El-Hajj C., Kyriacou P.A. (2020). A review of machine learning techniques in photoplethysmography for non-invasive cuff-less measurement of blood pressure. Biomed. Signal Process. Control.

[12] Chen Y., Wen C., Tao G., Bi M. (2012). Continuous and noninvasive measurement of systolic and diastolic blood pressure by one mathematical model with the same model parameters and two separate pulse wave velocities. Ann. Biomed. Eng..

[13] Kao Y.H., Chao P.C.P., Wey C.L. (2019). Design and validation of a new PPG module to acquire high-quality physiological signals for high-accuracy biomedical sensing. IEEE J. Sel. Top. Quantum Electron..

[14] Thambiraj G., Gandhi U., Mangalanathan U., Jose V.J.M., Anand M. (2020). Investigation on the effect of Womersley number, ECG and PPG features for cuff less blood pressure estimation using machine learning. Biomed. Signal Process. Control.

[15] Turgutkaya A., Asci G. (2021). The association between Hba1c and arterial stiffness among non-diabetic patients with chronic kidney disease. J. Vasc. Bras..

[16] Blancher J., Asmar R., Djane S., London G.M., Safar M.E. (1999). Aortic pulse wave velocity as a marker of cardiovascular risk in hypertensive patients. Hypertension.

[17] Parikh J.D., Hollingsworth K.G., Kunadian V., Blamire A., MacGowan G.A. (2016). Measurement of pulse wave velocity in normal aging: Comparison of vicorder and magnetic resonance phase contrast imaging. BMC Cardiovasc. Disord..

[18] Chan Y., Zhang D., Karimi H.R., Deng C., Yin W. (2022). A new deep learning framework based on blood pressure range constraint for continuous cuffless BP estimation. Neural Netw..

[19] Panwar M., Gautam A., Biswas D., Acharrya A. (2020). PP-Net: A deep learning framework for PPG based blood pressure and heart rate estimation. IEEE Sens. J..

[20] Sulochana C.H. (2021). A review of photoplethysmography based measurement of blood pressure and heart rate variability. J. Bioeng. Biomed. Sci..

[21] Zahedi E., Sohani V., Ali M.A.M., Chellappan K., Beng G.K. (2015). Experimental feasibility study of estimation of the normalized central blood pressure waveform from radial photoplethysmogram. J. Healthc. Eng..

[22] Jana B., Oswal K., Mitra S., Saha G., Banerjee S. (2020). Windkessel model-based cuffless blood pressure estimation using continuous wave doppler ultrasound system. IEEE Sens. J..

[23] Ding X., Yan B.P., Zhang Y.T., Liu J., Zhao N., Tsang H.K. (2017). Pulse transit time based continuous cuffless blood pressure estimation: A new extension and a comprehensive evaluation. Sci. Rep..

[24] Samimi H., Dajani H.R. Cuffless blood pressure estimation using cardiovascular dynamics. Proceedings of the International Conference on Electrical, Computer and Energy Technologies (ICECET 2022).

[25] Chao P.C.P., Wu C.C., Nguyen D.H., Nguyen B.S., Huang P.C., Le V.H. (2021). The machine learnings leading the cuffless PPG blood pressure sensors into the next stage. IEEE Sens. J..

[26] Bramwell J.C., Hill A.V. (1922). The velocity of the pulse wave in man. Proc. R. Soc. Lond. Biol. Sci..

[27] Brennan E.G., O’Hare N.J., Walsh M.J. (1998). Transventicular pressure-velocity wave propagation in diastole: Adherence to the Moens-Korteweg equation. Physiol. Meas..

[28] Liu D., Gorges M., Jenkins S.A. (2012). University of Queensland vital signs dataset: Development of an accessible repository of anesthesia patient monitoring data for research. Anesth. Analg..

[29] Pan J., Tompkins W.J. (1985). A real-time QRS detection algorithm. IEEE Trans. Biomed. Eng..

[30] Ramshur J.T. (2010). Design, Evaluation, and Application of Heart Rate Variability Analysis Software. Master’s Thesis.

[31] Shaffer F., Ginsberg J.P. (2017). An overview of heart rate variability metrics and norms. Front. Public Health.

[32] Thong T., McNames J., Aboy M. Lomb-Wech periodogram for non-uniform sampling. Proceedings of the 26th Annual International Conference of the IEEE EMBS.

[33] Clifford G.D., Tarassenko L. (2005). Quantifying errors in spectral estimates of HRV due to beat replacement and resampling. IEEE Trans. Biomed. Eng..

[34] Kamen P.W., Tonkin A.M. (1995). Application of the Poincaré plot to heart rate variability: A new measure of functional status in heart failure. Intern. Med. J..

[35] Richman J., Moorman J.R. (2000). Physiological time-series analysis using approximate entropy and sample entropy. Am. J. Physiol..

[36] Peng C.K., Buldyrev S.V., Havlin S., Simons M., Stanley H.E., Goldberger A.L. (1994). Mosaic organization of DNA nucleotides. Phys. Rev. E.

[37] McDuf D., Gontarek S., Picard R.W. (2014). Remote detection of photoplethysmographic systolic and diastolic peaks using a digital camera. IEEE Trans. Biomed. Eng..

[38] Bank A.J., Wilson R.F., Kubo S.H., Holte J.E., Dresing T.J., Wang H. (1995). Direct effects of smooth muscle relaxation and contraction on in vivo human brachial artery elastic properties. Circ. Res..

[39] Tseng T.J., Tseng C.H. (2020). Cuffless blood pressure measurement using a microwave near-field self-injection-locked wrist pulse sensor. IEEE Trans. Microw. Theory Tech..

[40] Wang H.S.J., Yeh M.H., Chao P.C.P., Tu T.Y., Kao Y.H., Pandey R. (2020). A fast chip implementing a real-time noise resistant algorithm for estimating blood pressure using a non-invasive, cuffless PPG sensor. Microsyst. Technol..

[41] Elgendi M., Liang Y., Ward R. (2018). Toward generating more diagnostic features from photoplethysmogram waveforms. Disease.

[42] Charlton P.H., Harana J.M., Vennin S., Li Y., Chowienczyk P., Alastruey J. (2019). Modeling arterial pulse waves in healthy aging: A database for in silico evaluation of hemodynamics and pulse wave indexes. Am. J. Physiol. Heart Circ. Physiol..

[43] Shao J., Shi P., Hu S., Yu H. (2020). A revised point-to-point calibration approach with adaptive errors correction to weaken initial sensitivity of cuff-less blood pressure estimation. Sensors.

[44] Hameed S.S., Petinrin O.O., Hashi A.O., Saeed F. (2018). Filter-wrapper combination and embedded feature selection for gene expression data. Int. J. Adv. Soft Comput. Its Appl..

[45] Yao Y., Rosasco L., Caponnetto A. (2007). On early stopping in gradient descent learning. Constr. Approx..

[46] Song X., Bryan B.A., Paul K.I., Zhao G. (2012). Variance-based sensitivity analysis of a forest growth model. Ecol. Model..

[47] Saltelli A., Annoni P., Azzini I., Campolongo F., Ratto M., Tarantola S. (2010). Variance based sensitivity analysis of model output. Design and estimator for the total sensitivity index. Comput. Phys. Commun..

[48] Pianosi F., Sarrazin F., Wagener T. (2015). A Matlab toolbox for global sensitivity analysis. Environ. Model. Softw..

[49] (2014). IEEE Standard for Wearable Cuffless Blood Pressure Measuring Devices.

[50] Mori H., Saito I., Eguchi E., Maruyama K., Kato T., Tanigawa T. (2014). Heart rate variability and blood pressure among Japanese men and women: A community-based cross-sectional study. Hypertens. Res..

[51] Takalo R., Korhonen I., Turjanmaa V., Majahalme S., Tuomisto M., Uusitalo A. (1994). Short-term variability of blood pressure and heart rate in borderline and mildly hypertensive subjects. Hypertension.

[52] Ribezzo S., Spina E., Di Bartolomeo S., Sanson G. (2014). Noninvasive techniques for blood pressure measurement are not reliable alternative to direct measurement: A randomized crossover train in ICU. Sci. World J..

[53] Kaur B., Kaur S., Yaddanapudi L.N., Singh N.V. (2019). Comparison between invasive and noninvasive blood pressure measurements in critically ill patients receiving inotropes. Blood Press. Monit..

[54] Malpas S.C. (2002). Neural influences on cardiovascular variability: Possibilities and pitfalls. Am. J. Physiol.—Heart Circ. Physiol..

